# Computational Insights into the Interaction between Cytoadherence Receptor gC1qR and the DBLβ12 Domain of a *Plasmodium falciparum* PfEMP1 Ligand

**DOI:** 10.3390/life11090993

**Published:** 2021-09-21

**Authors:** Rowaida Bakri, Mohd Rehan, Hina Shamshad, Abdul Hafiz

**Affiliations:** 1 College of Medicine, Umm AL Qura University, Makkah 21955, Saudi Arabia; rabakri@uqu.edu.sa (R.B.); henathebest@gmail.com (H.S.); 2 King Fahd Medical Research Center, King Abdulaziz University, Jeddah 21589, Saudi Arabia; 3 Department of Medical Laboratory Technology, Faculty of Applied Medical Sciences, King Abdulaziz University, Jeddah 21589, Saudi Arabia; 4 Epidemiology, London School of Hygiene and Tropical Medicine, University of London, London WC1E 7HT, UK

**Keywords:** malaria, cytoadherence, gC1qR, *Plasmodium falciparum* erythrocyte membrane protein 1 (PfEMP1), Duffy binding-like (DBL) domain

## Abstract

Human receptor gC1qR is a 32 kD protein that mediates the cytoadherence of *Plasmodium falciparum*-infected erythrocytes (IEs) to human brain microvascular endothelial cells (HBMEC) and platelets. The cytoadherence of IEs to gC1qR has been associated with severe malaria symptoms. The cytoadherence to gC1qR is mediated by the Duffy binding-like β12 (DBLβ12) domain of *Plasmodium falciparum* erythrocyte membrane protein 1 (PfEMP1), PFD0020c. Here, we report the structural insights into the binding of the DBLβ12 domain of PfEMP1 with the human receptor gC1qR using computational methods. A molecular model of the DBLβ12 domain was generated and used for protein–protein docking with the host receptor gC1qR. The protein–protein docking revealed that the DBLβ12 asymmetrically interacts with two subunits of the gC1qR trimer at the solution face of gC1qR. A total of 21 amino acid residues of DBLβ12 interact with 26 amino acid residues in the gC1qR trimer through 99 nonbonding interactions and 4 hydrogen bonds. Comparative analysis of binding sites on the DBL domain fold for the two receptors gC1qR and ICAM1 showed that the two sites are distinct. This is the first study that provides structural insights into DBLβ12 binding with its receptor gC1qR and may help in designing novel antisevere malaria interventions.

## 1. Introduction

Malaria is one of the most devastating parasitic diseases. Malaria has caused about 409,000 deaths in 2019. An estimated 67% of all malaria deaths are among children under 5 years of age [1]. Most of the malaria related deaths are caused by *Plasmodium falciparum* infections, although 5 species of *Plasmodium* are known to cause human malaria. The *P. falciparum* infected erythrocytes (IEs) have the unique ability to cytoadhere to host cells and completely sequester in the blood vasculature of the human host. The *P. falciparum*-infected erythrocytes may form rosettes and clumps when they cytoadhere to uninfected erythrocytes and platelets, respectively. The *P. falciparum*-infected erythrocytes can also bind and adhere to microvascular endothelial cells and sequester in the blood vasculature. Sequestration of *P. falciparum*-infected erythrocytes may lead to obstruction in blood flow, elevation in locally released cytokine levels, and local hypoxia. Sequestration in the brain and placenta has been linked to severe malaria. Cytoadherence is thus considered as an important virulence mechanism of *P. falciparum* [2,3]. Blocking or reversing the sequestration in the brain and placenta may provide novel interventions against cerebral- and pregnancy-associated malaria, respectively [4,5]. Therefore, the study of receptor–ligand interactions involved in the cytoadherence of *P. falciparum* is important for the research and development of novel malaria vaccines and antimalaria drugs.

Cytoadherence is mediated by specific receptor–ligand interactions. More than a dozen human receptors for cytoadherence have been identified, including CD36 [6], intracellular adhesion molecule-1 (ICAM1/CD54) [7], chondroitin sulphate-A (CSA) [8], endothelial protein C receptor (EPCR) [9], and gC1qR [10]. The parasite partner proteins in the receptor–ligand interaction pair mediating cytoadherence are known to be *P. falciparum* erythrocyte membrane protein 1 (PfEMP1) family members. PfEMP1 proteins are variant surface antigens of *P. falciparum* encoded by *var* genes [11]. About 60 *var* genes exist in a single *P. falciparum* genome, encoding just as many PfEMP1 proteins. Transcriptional switching of *var* gene expression provides a new cytoadherence phenotype to the parasite. The switching of *var* gene expression is a tool that allows the parasite to evade the host’s immune response through antigenic variation [12]. PfEMP1 family members follow mutually exclusive expression patterns; a single member is expressed on the surface of a mature stage *P. falciparum*-infected erythrocyte [13].

The PfEMP1 proteins are transmembrane proteins that are expressed on the surface of an infected erythrocyte. PfEMP1 proteins are encoded by *var* genes. The *var* genes have been classified into 5 groups (A, B, C, AB, and BC groups) based on chromosomal location, gene orientation, and sequence features. Studies have suggested that group A and group B/A *var* gene expression in the IEs isolated from human hosts may be associated with severe malaria [14]. The large extracellular domain of PfEMP1s have multiple adhesive domains, including Duffy binding-like (DBL) domains and cystine-rich interdomain region (CIDR) domains. Based on the organization of these adhesive domains within a PfEMP1 protein, PfEMP1s have been further classified into different domain cassettes [15]. 

Earlier, we identified a novel receptor gC1qR that mediates the cytoadherence of *P. falciparum*-infected erythrocytes to human brain microvascular endothelial cells and platelets [10]. Studies suggest that cytoadherence to gC1qR may play an important role in severe malaria pathogenesis [16,17]. gC1qR protein exists as a homotrimer protein. It has neither a transmembrane domain, nor a predicted Glycosylphosphatidylinositol (GPI) anchor sequence [18]. However, the protein is known to be expressed on the surface of several human cells, including human brain microvascular endothelial cells, dendritic cells, and platelets [10]. The gC1qR trimer has two distinct faces: the membrane face and the solution face [18]. In addition to the surface of human cells, gC1qR is also present in blood serum in soluble form [19]. Activated and proliferating cells secrete a soluble form of gC1qR (sgC1qR), which is similar to gC1qR in structure and function [20]. A conserved motif consisting of 174–180 amino acids of sgC1qR is essential for its binding to endothelial cells through surface bound fibrinogen. Endothelial cells bound sgC1qR can act in an autocrine and paracrine manner to facilitate vasodilation [21]. Interestingly, mature-stage *P. falciparum*-infected erythrocytes can bind to soluble gC1qR [22]. 

The malaria parasite ligand that mediates cytoadherence to gC1qR has been identified as the PFD0020c protein, a member of the PfEMP1 family belonging to group A *var* gene. The PfD0020c protein has 5 DBL domains and 2 CIDR domains in its extracellular adhesive domains. It is the DBLβ12 domain of PfD0020c that interacts with gC1qR [23]. However, the interface region and interacting residues of DBLβ12 have not been explored yet. Computational methods including sequence information theoretic methods [24,25] and molecular docking simulation methods [26,27] are useful in the study of functionally critical residues and interacting residues in receptor–ligand interactions. Here, we have investigated the interface region and the key interacting residues that mediate the binding of gC1qR with DBLβ12 using computational methods. 

## 2. Materials and Methods

Data Retrieval

The sequence of the PfEMP1 protein of *Plasmodium falciparum* (Strain 3D7) was obtained from the PlasmoDB database [28] by translating the transcript PFD0020c (New Transcript ID, PF3D7_0400400). The X-ray crystal structure of human receptor gC1qR in complex with Factor XII and kininogen with PDB Id: 6szw was obtained from the PDB database. This receptor structure was exploited for the protein–protein docking experiment.

Identification of DBLβ12 Domain in PfEMP1 Protein

For scanning Duffy binding domains, the PfEMP1 protein sequence was fed into the pfam database [29] and searched. The DBLβ12 domain was identified as ranging from 843–1030 amino acids of the PfEMP1 protein. However, we considered a broader range with additional residues at both ends, i.e., 745–1183 amino acids. This stretch of PfEMP1 was used for modeling of the DBLβ12 domain as this was the largest stretch encompassing the DBLβ12 domain matching its template in the PDB. The template was identified by the Blastp option of NCBI Blast.

Molecular Modeling of the DBLβ12 Domain

Molecular modeling of the DBLβ12 domain of PfEMP1 was carried out using Modeller [30]. The template used for modeling was an ICAM1 binding DBLβ from the PDB. We have used an uncomplexed structure of DBLβ (6s8t) for our modelling to avoid ICAM1 binding-induced conformational changes. A total of 100 models were prepared using Modeller. The five best models were chosen based on either the lower value of the Modeller objective function or a DOPE assessment score with a higher value than the GA341 assessment score. Finally, the single best model was selected by Ramachandran plot analysis of the five best models using Procheck [31].

Protein–Protein Docking

The molecular docking of the DBLβ12 domain and the human gC1qR was carried out using Patchdock [31], and the predicted complexes were further refined using Firedock [32]. The Patchdock algorithm uses shape complementary criteria for protein–protein docking. The two protein molecules were divided into patches (concave, convex, and flat patches) and then the patches of the two proteins were superimposed for possible match. Finally, the obtained protein–protein complexes were scored and ranked using a geometric shape complementarity score.

Analysis of Protein–Protein Complex

The visual analysis of the protein–protein complex was performed using Pymol (DeLano WL: PyMOL. San Carlos, CA. DeLano Scientific, 2002), and illustrations were prepared. The molecular interactions between the DBLβ12 domain and the human gC1qR were predicted using the Dimplot option of Ligplot+ [33].

Sequence Alignment Analysis

The analysis and final illustration preparation of sequence alignment was performed using Jalview [34].

Molecular Dynamic Simulation

Molecular dynamic simulation was carried out by Gromacs with a charmm36-feb2021 force field. The proteins were solvated in a cubic box with a simple point-charge water molecule, spc216. The solvation box was generated having proteins at the center with edge distance from the surface of the protein at 10 Å. The whole solvated systems were neutralized by addition of counter ions (Na^+^ and Cl^−^) followed by energy minimization using the steepest descent method. The periodic boundary conditions were implemented to avoid surface effects. The energy-minimized system was subjected to NVT and then NPT equilibration for 100 ps each. The equilibrated DBLβ12 protein and DBLβ12-gC1qR complexes were simulated for 100 ns.

## 3. Results

### 3.1. Molecular Model of DBLβ12 Domain

**Figure 1:**
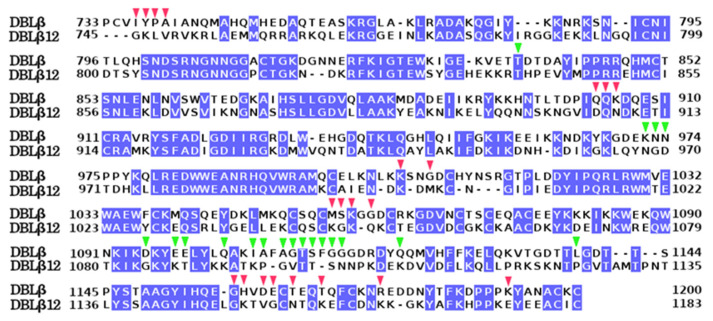
Sequence alignment of the model DBLβ12 with the template DBLβ (PDB ID: 6s8t). The identically conserved columns are highlighted in blue, and the conserved residues are shown in white color. The initial and final amino acid position in a row for the template and model are displayed, respectively. The red triangles mark the amino acid positions of DBLβ12 residues interacting with gC1qR, while the green triangles mark the positions of DBLβ residues interacting with ICAM1.

**Figure 2:**
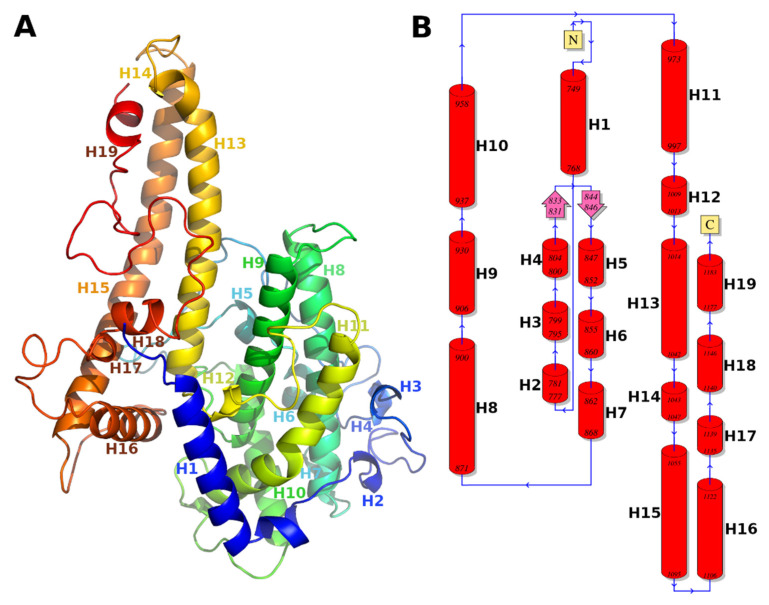
Molecular model of DBLβ12. (**A**) Ribbon representation of the DBLβ12 model colored in spectrum rainbow with helices labeled with helix number. Color is in gradient from the N-terminal to the C-terminal, starting from dark blue, continuing to green and then yellow and finally C-terminal in red. (**B**) Topology diagram of DBLβ12 domain with labeled helices. Topology diagram shows sequential secondary structure elements along the whole length of the protein. N and C enclosed in yellow squares represent N- and C- terminals of the protein. The red cylindrical structures marked with initial and final amino acid positions represent alpha helices, pink wide-arrows marked with initial and final amino acid positions represent beta strands, and blue lines marked with direction ‘N-terminal to C-terminal’ represent random coil or loops.

The gC1qR-binding DBLβ12 domain had 48% identity and 63% similarity with the template DBLβ that binds ICAM1 (PDB Id: 6s8t). The alignment of the model and template is shown in Figure 1, with identical residue columns highlighted in blue color. The structural model of the DBLβ12 domain is shown in ribbon representation colored rainbow spectrum and as a topology diagram in Figure 2. 

**Figure 3:**
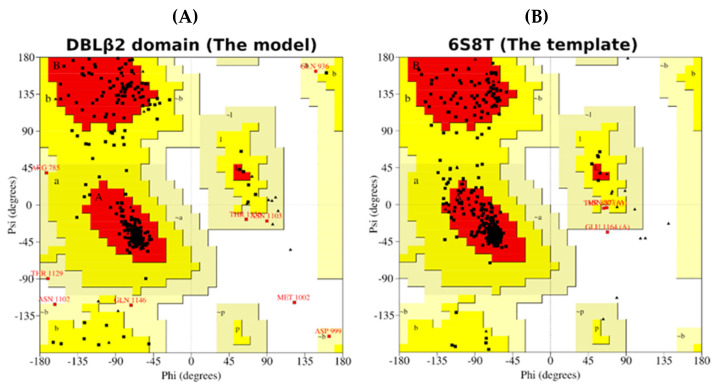
Ramachandran plot for (**A**) the model (DBLβ12) and (**B**) the template used. All the residues are shown as dots (except Glycine, shown as triangles) in four different regions of the plot: most favorable (A,B,L) in red color, additional allowed (a,b,l,p) in yellow, generously allowed (~a,~b,~l,~p) in light yellow, and disallowed regions (remaining) in white colors. Glycine residues shown as triangles are special in having no side chain (only H-atom), so restrictions for being in different regions of the plot does not apply on them.

The evaluation of the model using Ramachandran plot analysis is shown in Figure 3. Table 1 shows that a high percentage of residues, 87.3%, fall in the most favorable regions comparable to that of the template (89.0%). The low percentage of residues, 0.3% (only one residue), fall in disallowed regions, as seen in the template as well [35]. Ramachandran plot analysis establishes that the quality of the model is as good as the template.

### 3.2. Molecular Recognition of Human Receptor gC1qR by the DBLβ12 Domain

**Figure 4:**
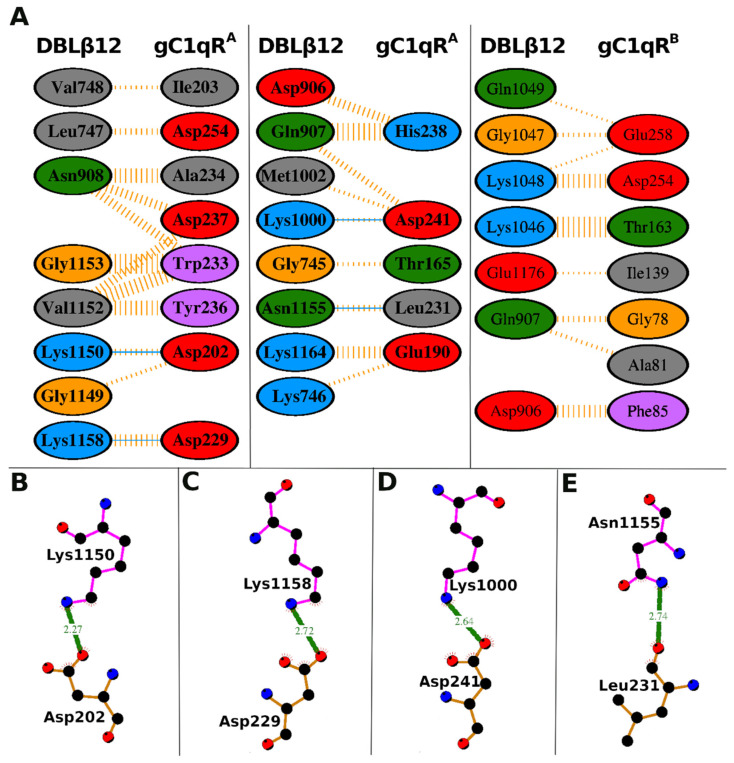
Protein–protein interaction plot of DBLβ12 and human receptor gC1qR. (**A**). The interacting proteins, DBLβ12 and gC1qR, are marked at the top, and their amino acid residues are shown in columns. The amino acids are shown in elliptical shapes and colored based on their physicochemical properties. The nonbonded contacts between two amino acids are shown as dashed lines in light orange color. The thickness of the dashed lines corresponds to the number of the interactions between the two residues. The hydrogen bonds between two residues are shown as blue lines. (**B**–**E**). The hydrogen bonds between DBLβ12 and the A subunit of gC1qR are shown in ball and stick representations. The balls represent atoms, and the lines joining two atoms represent bonds. The various atom types are distinguished based on color (C-atoms, black; N-atoms, blue; O-atoms, red). The hydrogen bonds are shown as green, thick lines labeled with bond length (Å). The residues with a pink backbone shown on the top of the hydrogen bond belong to DBLβ12, and the residues with a light orange backbone shown below the hydrogen bonds belong to A subunit of gC1qR.

Protein–protein docking analysis shows that the two proteins bind well, and the complementary surfaces, the projections, and the recesses in the surfaces were well interlocked. The docking results revealed that the DBLβ12 binds to the ligand accessible solution face of gC1qR and not to the membrane face. This agrees with what is expected as the solution face is the only face accessible for binding. The absolute value of the binding energy was high (−18.69 Kcal/mol), thus the complex formed was quite stable. The list of interacting residues for both the proteins and number of molecular interactions is provided in the Table 2. The protein–protein interaction plot for the interacting residues of both the proteins is shown in Figure 4.

### 3.3. Interface Area and Interacting Residues of DBLβ12-gC1qR Complex

DBLβ12 binds the trimeric gC1qR receptor by making interactions with the two identical monomers in the gC1qR trimer, as shown Figure 4 and Table 2. The three monomers of gC1qR in the gC1qR trimer are referred to as gC1qR^A^, gC1qR^B^, and gC1qR^C^, as per their subunit/monomer chain identifier (ID) in the PDB file. These monomers were identical, and the analysis was performed with respect to these monomer chain IDs. The monomer chain IDs were used here as references and showed that the DBLβ12 bound to the trimeric receptor and interacted differently with the two identical monomers in the trimer. Thus, the predicted DBLβ12 interaction was asymmetric with respect to the three monomers of human receptor gC1qR. The monomer gC1qR^A^ contributed most to the binding interactions by forming 62 nonbonded contacts and four hydrogen bonds with its DBLβ12 ligand. While the gC1qR^B^ was forming only 37 nonbonded contacts with its DBLβ12 ligand.

A total of 21 DBLβ12 residues were predicted to interact with gC1qR. These 21 interacting residues were Gly-745, Lys-746, Leu-747, Val-748, Asp-906, Gln-907, Asn-908, Lys-1000, Met-1002, Lys-1046, Gly-1047, Lys-1048, Gln-1049, Gly-1149, Lys-1150, Val-1152, Gly-1153, Asn-1155, Lys-1158, Lys-1164, and Glu-1176. These 21 DBLβ12 residues contributed to a total of 99 nonbonded contacts and four hydrogen bonds and stabilized the protein–protein complex. Among these 21 interacting residues of DBLβ12, four residues (Lys-1000, Lys-1150, Asn-1155, and Lys-1158) formed one hydrogen bond each. The maximum number of nonbonded interactions were 12, formed by Lys-1046.

The 21 DBLβ12 residues can be mapped to five continuous peptide stretches to facilitate the identification of targets to disrupt gC1qR-DBLβ12 interaction. These amino acid stretches include 745–748 (Peptide-1, 4 residues), 906–908 (Peptide-2, 3 residues), 1000–1002 (Peptide-3, 3 residues), 1046–1049 (Peptide-4, 4 residues), and 1149–1176 (Peptide-5, 28 residues). The peptide-1, peptide-3, and peptide-5 bind subunit gC1qR^A^. The Peptide-4 binds subunit gC1qR^B^. The peptide-2 binds both the subunits gC1qR^A^ and gC1qR^B^.

### 3.4. Comparative Analysis of gC1qR-Binding Site and ICAM1 Binding Sites in DBL Domains

**Figure 5:**
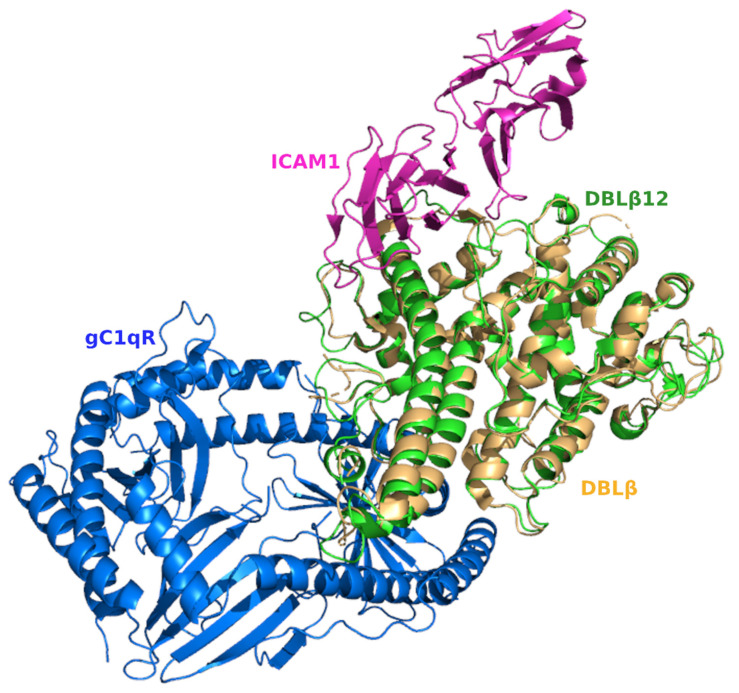
Structural superposition of DBL domains with bound respective receptors. All the protein subunits are shown in ribbon representation. The two DBL domains DBLβ12 and DBLβ are superimposed and colored green and light orange, respectively. The DBLβ receptor ICAM1 is shown in purple color, while the DBLβ12 receptor gC1qR is shown in blue color. The binding sites for the two receptors are located at different regions on the surface of the DBL domain.

The structural superposition of DBLβ bound with ICAM1 and DBLβ12 bound with gC1qR shows that the ICAM1 and gC1qR-binding site regions fell at two separate locations of the structural fold of the DBLβ domain, as shown in Figure 5. Thus, the interaction occurs at two distinct sites in the DBL domain with no overlapping regions.

The DBLβ domain that specifically binds ICAM1 and the DBLβ12 domain that binds gC1qR share 48% sequence identity. However, the sequence identity at the interacting sites of these two domains with their respective receptors was low, suggesting binding specificity induced selection pressure at these amino acid locations. Among the 21 DBLβ12 residues that interact gC1qR, only four residues (19%) were conserved in the alignment of DBLβ12 and DBLβ (red marked in Figure 1). Similarly, among 19 residues that interact ICAM1, only two residues (10%) were conserved in the alignment of DBLβ12 and DBLβ (green marked in Figure 1). These observations suggest that the receptor binding specificity is determined by the variations in the interacting residues of the DBLβ and DBLβ12 domains.

### 3.5. Molecular Dynamic Simulation

The molecular dynamic simulation was performed on the DBLβ12 model and the DBLβ12-gC1qR complex to evaluate the stability of the protein model and the complex, respectively. The DBLβ12 model was subjected to 100 ns simulation, and the RMSD plot showed the initial increase in RMSD (till approx. 16 ns), and then the plateau was reached. The low deviation in RMSD values during 100 ns simulation indicate the good stability of the DBLβ12 model. The complex of the DBLβ12-gC1qR proteins was also subjected to 100 ns simulation, and the RMSD plot showed initial increase till 40ns, and then the plateau was reached. This observation of low deviations in RMSD values of the complex during the simulation also suggests that the protein complex is stable.

## 4. Discussion

This study has identified and characterized molecular interaction between gC1qR and DBLβ12 of PfEMP1 PFD0020c of *P. falciparum* 3D7 using computational methods. This is the first study to characterize the structural details of gC1qR–DBLβ12 interaction in malaria. Our study has used several rational approaches that have made our computational characterization more realistic. Firstly, to build the model of DBLβ12, we have used a template of another PfEMP1 DBLβ crystal structure in an unbound state to its receptor, and thus we have removed the influence of the bound receptor on the ligand in our model. Secondly, the identity and similarity between our model and the template protein sequence was 48% and 63%, respectively, giving us a fair amount of confidence in our predicted structure. Thirdly, the length of the amino acid sequence of the model was chosen to match the length of the template sequence, meaning we have included adjacent stretches of protein that may have influence on the folding of the DBLβ12 domain in the template while retaining all the conserved residues that are hallmarks of DBL domain. Fourthly, the amino acid residues that interact with gC1qR were not much conserved between the DBLβ12 and DBLβ despite a 48% sequence identity in the two ligands. The amino acid residues in the two DBL domains ought to be different since the structure of their receptors are different. And finally, while modeling the interaction, we have not given any constrains to the computer program to choose any portion or face of the receptor or ligand. Despite using such criteria, our model showed that the DBLβ12 binds to the solution face of gC1qR. The solution face is the only face of membrane attached gC1qR that is available for interaction to ligands.

**Figure A1:**
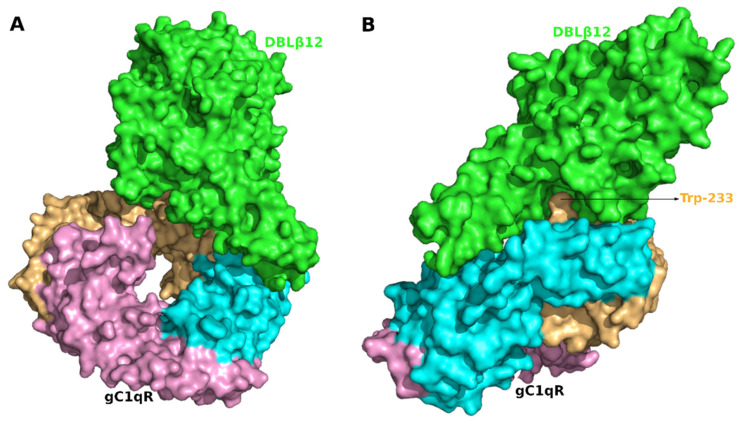
The complex of DBLβ12 and human receptor gC1qR in surface representation. (**A**). One view of the complex showing labeled DBLβ12 in green color and the trimeric receptor with each subunit colored differently. (**B**). Another view of the complex with interlocked surfaces between the DBLβ12 and the subunits of gC1qR. The position of conspicuous residue Trp-233 that forms the maximum nonbonding interactions with the ligand is shown.

Interestingly, we found that the two monomers, gC1qR^A^ and gC1qR^B^, of the gC1qR trimer interact with the DBLβ12 domain. This interaction is asymmetric with respect to the gC1qR trimer (Figure A1A) in Appendix A. This type of asymmetric interaction is expected when the receptor is multimeric and the ligand is a monomer. In fact, the interaction between gC1qR with factor XII and kininogen (PDB Id: 6szw) is asymmetric as well, involving only one subunit of gC1qR [36].

Protein–protein docking results showed that 21 of the DBβ12 residues interacted with 26 residues of the gC1qR trimer through 99 nonbonding interactions and 4 hydrogen bonds. Of the 21 interacting residues of DBLβ12, five residues, Lys-1000, Lys-1150, Asn-1155, Lys-1158, and Lys-1046, were proposed as key interacting residues since they formed the major part of the molecular interactions. We have identified 5 stretches of peptides in DBLβ12 that can be targeted for the disruption of gC1qR-DBLβ12 interactions. We have named these peptide stretches as peptide-1 to peptide-5 to facilitate identification. While peptide-1 to peptide-4 are three to four amino acid long, the peptide-5 is the longest, comprising 28 amino acids. Peptide-5 can be divided into two, since synthesis of smaller peptides is less error prone. These peptide regions can be targeted for disrupting gC1qR-DBLβ12 interactions. The monomer A of the gC1qR trimer makes most of the contacts with DBLβ12, including all 4 hydrogen bonds. Therefore, we anticipate that disrupting the interactions between DBLβ12 and gC1qR^A^ will have stronger disruptive effect on the receptor–ligand interactions as compared to targeting the interactions between DBLβ12 and gC1qR^B^. Disruption of the interactions between DBLβ12 and both gC1qR^A^ & gC1qR^B^ may have synergistic effects. Further studies are needed to test these predictions. The information of key interacting residues and the proposed amino acid stretches may help in research on the development of an anti-severe malaria vaccine as well as the rational designing of novel therapeutics to treat and manage severe malaria.

The DBLβ domain that binds ICAM1 and the DBLβ12 domain that binds gC1qR share sequence homology and belong to same domain class. It is intriguing how *P. falciparum* employs the different DBL domains to bind a variety of host receptors [37]. We attempted to understand the binding specificity of these two DBL domains. The structural superposition of the DBL domains and amino acid conservation analysis of the binding sites of ICAM1 and gC1qR for DBL domains (DBLβ and DBLβ12) revealed that the ICAM1 and gC1qR bind at distinct locations on the DBL domain surface, and the binding specificity may be attributed to variations in the amino acid positions found in the binding site regions for the two receptors despite them having a similar structure fold of the DBL domain.

Multiligand receptors like gC1qR generally employ certain residues more frequently to interact with their ligands. For instance, the hepatitis C virus core protein interacts with gC1qR at residues 188–259 [38], a site that overlaps with the DBLβ12 interacting site identified in this study. Similarly, we have found that Trp-233 is a very prominent amino acid that makes 17 nonbonded interactions with the DBLβ12. The Trp-233 has a conspicuous position on the solution face of the gC1qR trimer (Figure A1B), and it has also been reported to be involved in the interaction of gC1qR with kininogen and the generation of bradykinin as well [39]. It will be interesting to see if the binding of DBLβ12 to gC1qR can inhibit kininogen binding and bradykinin production.

The complement component C1q binds at residues 76–93 of gC1qR [40], a site containing three residues (Gly-78, Ala-81, and Phe-85) which interact with DBLβ12. Further, C1q and PfEMP1 proteins are very large in size. Therefore, the binding of one of the proteins to gC1qR is likely to hinder the binding of the other protein. If this happens, the cytoadherence of *P. falciparum*-infected erythrocytes to gC1qR may affect the localized complement activation.

To our knowledge, this is the first study that reports the interaction between gC1qR and its pathogen ligand involving two of its three subunits. This asymmetric interaction between gC1qR and the DBLβ12 domain employs several amino acid residues that gC1qR uses to interact with its other ligands as well [38,40]. However, Ile-139 and Thr-163 seems to be the gC1qR residues that are unique to its interaction with the malaria ligand DBLβ12. gC1qR is also secreted from human cells and is also present as a soluble receptor in the blood. Serum levels of soluble gC1qR get upregulated during inflammation and certain cancers. gC1qr has been implicated in cancer progression as an inducer of angiogenesis and facilitator of metastasis, as well as through the inhibition of innate immune response against cancerous cells through free C1q depletion [41]. Thus, gC1qR is considered as a potential therapeutic target against certain cancers [42]. Our previous works have shown that *P. falciparum*-infected erythrocytes bind to cell surface gC1qR as well as soluble gC1qR [10,16,22]. It will be interesting to see if during falciparum infection *P. falciparum*-infected erythrocytes bind to soluble gC1qR present in blood and modulate its local concentration and function. We hypothesize that gC1qR ligands of *P. falciparum* may provide valuable intervention targets, not only against severe malaria, but also against certain cancers. In fact, evidence suggests that another *P. falciparum* cytoadherence receptor, VAR2CSA, is useful for targeting human cancers [43]. Further studies are needed to explore the role of this important cytoadherence receptor in severe malaria and other diseases.

## Figures and Tables

**Table 1:** Ramachandran plot analysis of the model with respect to its template. The percentage of residues are listed in most favorable, additional allowed, generously allowed, and disallowed regions of the plot.

Protein	Status of the Residues in Ramachandran Plot			
	Most Favourable	Additional Allowed	Generously Allowed	Disallowed
Template	89.0%	10.3%	0.5%	0.3%
Model	87.3%	10.4%	2.0%	0.3%

**Table 2:** Interacting residues of the PfEMP1 DBLβ12 domain and homotrimeric receptor gC1qR at the interface area with number of nonbonded and hydrogen-bonding interactions. The gC1qR residues are marked with the name of monomeric subunit in parentheses (A) and (B).

DBLβ12 Residues	gC1qR Residues	Hydrogen Bonds	Non-Bonded Contacts
Gly-745	Thr-165 (A)		1
Lys-746	Glu-190 (A)		1
Leu-747	Asp-254 (A)		2
Val-748	Ile-203 (A)		1
Asp-906	His-238 (A)		3
Gln-907	His-238 (A)		6
Gln-907	Asp-241 (A)		2
Asn-908	Asp-237 (A)		3
Asn-908	Trp-233 (A)		3
Asn-908	Ala-234 (A)		5
Lys-1000	Asp-241 (A)	1	1
Met-1002	Asp-241 (A)		1
Gly-1149	Asp-202 (A)		1
Lys-1150	Asp-202 (A)	1	2
Val-1152	Trp-233 (A)		5
Val-1152	Tyr-236 (A)		5
Val-1152	Asp-237 (A)		3
Gly-1153	Trp-233 (A)		7
Asn-1155	Leu-231 (A)	1	2
Lys-1158	Asp-229 (A)	1	4
Lys-1164	Glu-190 (A)		4
Asp-906	Phe-85 (B)		7
Gln-907	Gly-78 (B)		3
Gln-907	Ala-81 (B)		2
Lys-1046	Thr-163 (B)		12
Gly-1047	Glu-258 (B)		2
Lys-1048	Glu-258 (B)		1
Lys-1048	Asp-254 (B)		8
Gln-1049	Glu-258 (B)		1
Glu-1176	Ile-139 (B)		1

**Figure A2:**
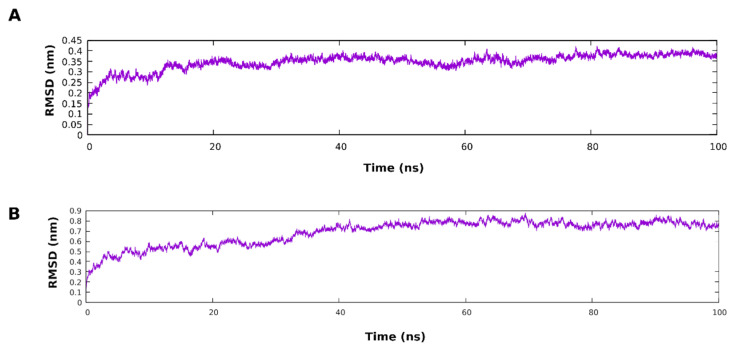
The molecular dynamic simulations of the DBLβ12 model and DBLβ12-gC1qR complex. (**A**) Root mean square deviation (RMSD) variation with respect to time for simulation of DBLβ12. (**B**). RMSD variation with respect to time for simulation of DBLβ12-gC1qR complex.

## Data Availability

The data presented in this study are available on request from the corresponding author.
